# Beta-synemin expression in cardiotoxin-injected rat skeletal muscle

**DOI:** 10.1186/1471-2474-8-40

**Published:** 2007-05-10

**Authors:** Yuji Mizuno, Jeffrey R Guyon, Akiko Ishii, Sachiko Hoshino, Norio Ohkoshi, Akira Tamaoka, Koichi Okamoto, Louis M Kunkel

**Affiliations:** 1 Department of Neurology, Gunma University Graduate School of Medicine, 3-39-22 Showa, Maebashi, Gunma 371-8511, Japan; 2 Howard Hughes Medical Institute/Division of Genetics, Children's Hospital Boston and Harvard Medical School, 300 Longwood Avenue, Boston, Massachusetts 02115, USA; 3 Department of Neurology, Institute of Clinical Medicine, University of Tsukuba, 1-1-1 Tennoudai, Tsukuba 305-8575, Japan; 4 Tsukuba University of Technology, Faculty of Health Science, Department of Neurology, 4-12-7 Kasuga, Tsukuba 305-8521, Japan

## Abstract

**Background:**

β-synemin was originally identified in humans as an α-dystrobrevin-binding protein through a yeast two-hybrid screen using an amino acid sequence derived from exons 1 through 16 of α-dystrobrevin, a region common to both α-dystrobrevin-1 and -2. α-Dystrobrevin-1 and -2 are both expressed in muscle and co-localization experiments have determined which isoform preferentially functions with β-synemin *in vivo*. The aim of our study is to show whether each α-dystrobrevin isoform has the same affinity for β-synemin or whether one of the isoforms preferentially functions with β-synemin in muscle.

**Methods:**

The two α-dystrobrevin isoforms (-1 and -2) and β-synemin were localized in regenerating rat tibialis anterior muscle using immunoprecipitation, immunohistochemical and immunoblot analyses. Immunoprecipitation and co-localization studies for α-dystrobrevin and β-synemin were performed in regenerating muscle following cardiotoxin injection. Protein expression was then compared to that of developing rat muscle using immunoblot analysis.

**Results:**

With an anti-α-dystrobrevin antibody, β-synemin co-immunoprecipitated with α-dystrobrevin whereas with an anti-β-synemin antibody, α-dystrobrevin-1 (rather than the -2 isoform) preferentially co-immunoprecipitated with β-synemin. Immunohistochemical experiments show that β-synemin and α-dystrobrevin co-localize in rat skeletal muscle. In regenerating muscle, β-synemin is first expressed at the sarcolemma and in the cytoplasm at day 5 following cardiotoxin injection. Similarly, β-synemin and α-dystrobrevin-1 are detected by immunoblot analysis as weak bands by day 7. In contrast, immunoblot analysis shows that α-dystrobrevin-2 is expressed as early as 1 day post-injection in regenerating muscle. These results are similar to that of developing muscle. For example, in embryonic rats, immunoblot analysis shows that β-synemin and α-dystrobevin-1 are weakly expressed in developing lower limb muscle at 5 days post-birth, while α-dystrobrevin-2 is detectable before birth in 20-day post-fertilization embryos.

**Conclusion:**

Our results clearly show that β-synemin expression correlates with that of α-dystrobrevin-1, suggesting that β-synemin preferentially functions with α-dystrobrevin-1 *in vivo *and that these proteins are likely to function coordinately to play a vital role in developing and regenerating muscle.

## Background

Synemin is a muscle intermediate filament protein that was originally identified in chickens [1]. Recently, human α- and β-synemin orthologues have been cloned [2,3], the latter of which was previously termed human desmuslin [2]. Both human synemin isoforms derive from the same gene as a result of differential splicing between exons 4 and 5 such that the β-synemin protein is 312 amino acids shorter at its C-terminus [4]. Both the β-synemin mRNA and protein are highly expressed in skeletal and cardiac muscle, while northern blot analysis also shows a weak doublet in brain [2], indicating that there are at least two different synemin isoforms expressed in that tissue. In humans, α-synemin is expressed in astrocytes of the optic nerve and in non-myelin-forming Schwann cells [5]. β-synemin was originally isolated in humans as an α-dystrobrevin- and desmin-interacting protein [2], two proteins expressed in differentiated muscle cells. Subsequent immunohistochemical analysis has shown that β-synemin localizes in human skeletal muscle to the costamere, the neuromuscular and myotendinous junctions, the Z-lines, and along the sarcolemma [2,4]. Although 12 different amino acid altering single-nucleotide polymorphisms have been identified within β-synemin's coding region, no causative mutations have yet been linked to a disease [6]; however, this gene is still a good disease candidate for myopathies of unknown etiology.

α-Dystrobrevin is one of the components of the dystrophin-associated protein complex (DAPC) [7] and interacts specifically with dystrophin and syntrophin in skeletal muscle [8]. The DAPC is thought to function as a structural link between the extracellular matrix and the internal cytoskeleton, although recently there has been speculation that the complex may also be involved in some type of signaling pathway. Interestingly, neuronal nitric oxide synthase (nNOS) levels have been shown to be significantly reduced in α-dystrobrevin-deficient muscle [9]. Through alternative splicing, α-dystrobrevin is expressed in several isoforms with α-dystrobrevin-1, -2, and -3 being the most highly expressed in skeletal muscle [7]. α-Dystrobrevin-1 is the largest isoform and has a unique 189 amino acid C-terminus, whereas α-dystrobrevin-2 is slightly smaller and contains a unique 16 amino acid C-terminus. The amino acid sequence originally used as the two-hybrid "bait" to isolate β-synemin was a sequence shared between α-dystrobrevin-1 and -2 [2]. It is therefore possible that both types of α-dystrobrevin interact with β-synemin, although one isoform of α-dystrobrevin might preferentially interact with β-synemin *in vivo*.

Recently, Hoshino et al. examined the expression of many DAPC proteins in regenerating rat tibialis anterior muscle following cardiotoxin injection [10-13]. Using western blot analysis, they found that β-dystroglycan was expressed very early during muscle regeneration and reached half-maximal expression within 1 day following cardiotoxin injection [11]. Dystrophin was first detected at day 3 and reached half-maximal expression by day 5.3 [10-13]. α-Sarcoglycan reached half-maximal expression at day 4.3 [11], α1-syntrophin at day 6.0 [10], α-dystrobrevin-1 at day 6.6 [12], and nNOS at day 11.7 [13]. This data suggested that protein reexpression during muscle regeneration occurred in an ordered fashion based on protein location. For example, proteins expressed within the basement membrane were expressed earlier than subsarcolemmal proteins, although even there, structural proteins (like dystrophin) were expressed earlier than proteins associated with signaling (such as α1-syntrophin, α-dystrobrevin-1, and nNOS). Expression data for β-synemin and α-dystrobevin-2 was not reported.

To better understand the role of β-synemin in muscle, we used co-immunoprecipitation, co-localization, and time-course experiments to monitor the expression of β-synemin and α-dystrobrevin in regenerating and developing rat tibialis anterior muscle. By correlating β-synemin expression with the reported expression patterns for dystrophin and its associated proteins [10-13], it is possible to start to dissect the function of β-synemin in muscle. Specifically, we correlate β-synemin expression with α-dystrobrevin-1 and -2, two potential β-synemin-interacting proteins. Our experiments show that β-synemin expression highly correlates with that of α-dystrobrevin-1 in developing and regenerating muscle, suggesting that β-synemin is more likely to coordinately function with α-dystrobrevin-1 than α-dystrobrevin-2 *in vivo*.

## Methods

### Animal care

Male Wistar rats (7 weeks old, 170–190 g, n = 48) were obtained from CLEA Japan, Inc., Tokyo. For the duration of the experiment, the rats were housed with 2 to 3 animals per cage and were kept at a constant temperature of 25°C with a 12-hour-light/dark cycle. All animal protocols required for this study were approved by the Institutional Animal Welfare Committee of the Animal Experiment Center at the University of Tsukuba. Female Wistar rats pregnant with 16-day-old and 18-day-old unborn embryos and female Wistar rats with 1-day-old, 3-day-old, 5-day-old, and 7-day-old pups were obtained from CLEA Japan, Inc., Tokyo.

### Immunoprecipitation

Protein extracts from rat tibialis anterior muscle taken 21 days after cardiotoxin injection were prepared by homogenization in 10 volumes of ice-cold RIPA buffer, pH 7.4, containing freshly added protease inhibitors (0.1 mM PMSF, 1 mg/ml each of antipain, leupeptin, aprotinin, and pepstatin A) followed by centrifugation at 1,000xG for 10 minutes at 4°C. 200 μl aliquots of extract were used for each immunoprecipitation. 15 μl of either a rabbit polyclonal anti-β-synemin antibody [4] or a mouse monoclonal anti-α-dystrobrevin antibody (BD Transduction Laboratories, Lexington, KY, USA) and 50 μl of 50% Protein G Sepharose 4 Fast Flow (GE Healthcare, Tokyo, Japan) were added as indicated and incubated for 16 hours at 4°C with rotation. Immunoprecipitants were separated by sodium dodecylsulfate (SDS)-polyacrylamide gel electrophoresis, and the gel was probed by western blotting with either the anti-α-dystrobrevin antibody or the anti-β-synemin antibody as indicated.

### Cardiotoxin injection

Animals were temporarily anesthetized with an inhalation of diethyl ether and an intraperitoneal injection containing pentobarbital sodium (60 mg/kg). For assaying muscle recovery, a single dose of cardiotoxin (1 ml of a 2 μM stock) from the Taiwan cobra, Naja naja atra (Latoxan, Valence, France), was injected intramuscularly into the right tibialis anterior muscle of 24 rats. As a control, the contralateral muscles were injected in the same animals with sterile 0.9% saline. After injection, the animals were returned to their cages and allowed free access to food and water.

### Muscle sampling

Rats were anesthetized using diethyl ether inhalation and a subsequent intraperitoneal injection of pentobarbital sodium (60 mg/kg). After removal of their injected muscles, the rats were euthanized with an intraperitoneal injection of an overdose of pentobarbital sodium. For examination, cardiotoxin-injected muscles were removed from each of the 9 tibialis anterior muscles on days 1, 3, 5, 7, 10, 14, 21 and 28 post-injection.

### Preparation of muscles for histochemical and immunohistochemical analyses

Biopsied muscles were immersed in OCT and frozen in chilled isopentane cooled by liquid nitrogen. Serial frozen sections were prepared at 10 μm and used for immunohistochemical and ATPase staining. For immunohistochemical analysis, sections were blocked in a solution supplied in the ABC-kit (avidin-biotinylated enzyme complex method; Vector Laboratories, Burlingame, CA, USA) for 20 minutes at room temperature and incubated overnight at 4°C with the anti-β-synemin antibody [4] diluted 1:1000 in hybrization solution provided in the ABC-kit. The slides were washed 3 times for 10 minutes each in ice-cold 1xPBS and then incubated with a donkey anti-rabbit Cy3-conjugated secondary antibody (Jackson ImmunoResearch Laboratories, West Grove, PA, USA) for 1 hour at room temperature. The slides were washed 3 times for 10 minutes each in ice-cold 1xPBS before mounting in vectashield. The slides were analyzed using an Axiophoto 2 (Carl Zeiss, Oberkochen, Germany) fluorescent microscope.

For confocal analysis of β-synemin and α-dystrobrevin, sections were prepared from rat tibialis anterior muscle sectioned 21 days after cardiotoxin injection. Sections were incubated overnight at 4°C with a mix of the anti-β-synemin antibody and the anti-α-dystrobrevin antibody (BD Transduction Laboratories), an antibody that recognizes both the type 1 and type 2 α-dystrobrevin isoforms. The slides were washed 3 times for 10 minutes each in ice-cold 1xPBS and then incubated with a mix of a donkey anti-rabbit Cy3-conjugated secondary antibody (Jackson ImmunoResearch Laboratories) for β-synemin and a goat anti-mouse IgG-biotin followed by Streptavidin-fluorescein (Amersham Pharmacia Biotech, Buckinghamshire, UK) for α-dystrobrevin. After washing in 1xPBS the sections were mounted in 90% glycerol in 1xPBS. A Leica DM IRB with TNC-NT confocal scanning laser microscope (Leica TCS SP/DM IRB inverted confocal scanning microscope; Leica Microsystems GmbH, Wetzlar, Germany) was used for the confocal microscopy recordings.

For ATPase analysis, serial sections were preincubated at pH 4.3, 4.6, and 10.4, respectively. Then, sections were incubated in ATP solutions (75 mg ATP disodium salt in 9.47 mM calcium chloride and 21 mM sodium barbital) for 25 minutes at room temperature. After washing with 1% calcium chloride, the slides were incubated in 2% cobalt chloride for 10 minutes and developed in a 1% ammonium sulfide solution for 30 seconds following washing in 0.1 M sodium barbital solutions. After dehydration in ascending alcohols (50%, 70%, 80%, 95%x2, 100%x2), slides were mounted with canada balsam.

### Immunoblot analysis

10 μm cross-sections of cardiotoxin-injected tibialis anterior muscle were stained with hematoxylin and eosin to assay for degenerating or regenerating fibers. For electrophoretical analysis, cardiotoxin affected tissue was physically excised from the section using a scalpel and protein extracts were prepared (total amount: 20–50 mg) by homogenizing excised tissue fragments in 20 volumes of ice-cold RIPA buffer (pH 7.4) containing freshly added protease inhibitors (0.1 mM PMSF, 1 mg/ml each of antipain, leupeptin, aprotinin, and pepstatin A). Insoluble particles were removed by centrifugation at 1000xG for 10 minutes at 4°C. Protein concentration was measured using a bicinchoninic acid protein assay (Pierce, Rockford, IL, USA). 5 μl of each extract (4.0 mg protein/ml) was separated on a 10% sodium dodecylsulfate-polyacrylamide gel electrophoresis (SDS-PAGE). Proteins were transferred to Hybond-P membranes (Amersham Pharmacia Biotech) in transfer buffer (25 mM Tris/192 mM glycine/20% v/v methanol, pH 8.3) at 30 V for 16 hours using a Trans-Blot Cell apparatus (Bio Rad Laboratories, Hercules, CA, USA). Membranes were blocked with 5% w/v skimmed dried milk powder in Tris buffered saline (20 mM Tris, 137 mM NaCl, pH 7.6) plus 0.1% Tween 20 (TBST) for 1 hour at room temperature, washed three times with TBST, and probed for 2 hours with either the anti-β-synemin [4] or the anti-α-dystrobrevin (1:200 dilution, BD Transduction Laboratories) primary antibody. Membranes were washed three times with TBST and then incubated for 1 hour with horseradish peroxidase (HRP)-conjugated donkey anti-rabbit IgG (H + L) secondary antibody for β-synemin staining or horseradish peroxidase (HRP)-linked sheep anti-mouse whole IgG secondary antibody for α-dystrobrevin staining (GE Healthcare, Tokyo, Japan). The membrane was washed in TBST and the HRP-conjugated protein was detected by ECL according to the manufacturers instructions (Amersham Pharmacia Biotech). To verify that each lane was loaded with equal amounts of protein (see above for protein quantification), the intensity of non-specific background bands was compared in the immunoblots being analyzed.

To assay protein expression during development, crude muscle was obtained from the whole lower limb of rats at embryonic day 16 and 18, whole distal lower limb muscles were isolated at embryonic day 20 and 1 day-old rats, and partial back portions of distal lower limb muscles were prepared from 3-, 5-, and 7-day-old and adult rats. Excised muscles were weighed and suspended in 20 volumes (w/v) of loading buffer containing 70 mM Tris-HCl (pH 6.7), 10% SDS, 10 mM ethylenediamine tetraacetic acid, and 5% β-mercaptoethanol. Suspensions were boiled for 5 minutes, left at room temperature for 10 minutes, and clarified by centrifugation at 15,000 rpm for 10 minutes at room temperature. Proteins were separated by SDS-PAGE on 5–15% acrylamide gels (Bio Craft, Tokyo, Japan), transferred to Hybond-P membranes (Amersham Pharmacia Biotech) in transfer buffer (48 mM Tris/39 mM glycine/13 mM SDS/20% methanol) at 15 V for 20 minutes using a Trans-Blot SemiDry apparatus (Bio Rad Laboratories), and blocked with blocking buffer (0.1% gelatin/0.1% casein/1xPBS) for 1 hour at 4°C. Blots were probed for 2 hours simultaneously with antibodies against β-synemin (1:1,000) and α-dystrobrevin (1:2,000). Membranes were washed with 0.1% Tween 20/1xPBS for 1 hour, and then incubated for 1 hour with a mix of horseradish peroxidase (HRP)-conjugated donkey anti-rabbit IgG (H + L) and anti-mouse IgG (H + L) secondary antibodies (Jackson ImmunoResearch Laboratories) in blocking buffer with 0.1% Tween 20. The membrane was washed in 0.1% Tween 20/1xPBS for 1 hour and the HRP-conjugated protein was detected using ECL+plus reagent (Amersham Pharmacia Biotech). To verify that each lane was loaded with equal amounts of protein, the intensity of non-specific background bands was compared in the immunoblots being analyzed.

### Quantitation of immunoblots

Densitometry was used to obtain quantitative data from western blots. HyperfilmECL film (Amersham Pharmacia Biotech) was scanned at an optical resolution of 400 dpi using an Epson GT9500 flatbed scanner. Densitometric analysis for each blot was performed with a computerized image processing system (NIH image 1.62). Previous experiments have shown this system yields densitometry measurements that are linear with respect to the amount of protein analyzed [10-13]. The relative protein levels on days 1 and 28 were set at 0% and 100%, respectively. For each of the other days, the relative protein levels were determined based on the density of the band relative to that on day 28. The mean relative protein level + SD on each day (n = 6–9) was plotted on sequential lines.

## Results

**Figure 1:**
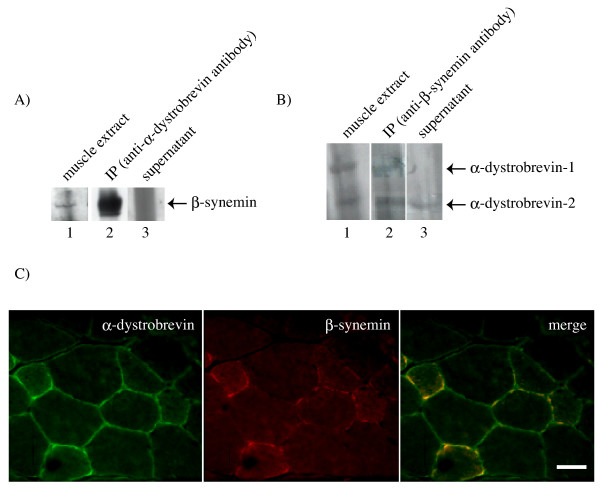
β-synemin and α-dystrobrevin interact *in vivo *and co-localize in rat muscle. (A) Using an anti-α-dystrobrevin antibody, α-dystrobrevin and its interacting proteins were immunoprecipitated from rat protein muscle extracts. Western blot analysis was performed to localize the β-synemin protein. β-synemin was found to be present in the muscle extract (1) and immunoprecipitated pellet (2), while it was not detected in the supernatant (3). (B) Using an anti-β-synemin antibody, β-synemin and its interacting proteins were immunoprecipitated from rat protein muscle extracts. Western blot analysis was performed to localize the α-dystrobrevin isoforms. Both α-dystrobrevin-1 and -2 were detected in the muscle extract (1) and pellet (2), while only α-dystrobrevin-2 was present in the supernatant (3). (C) Rat tibialis anterior muscle was sectioned 21 days following cardiotoxin injection and double immunostained with antibodies against α-dystrobrevin antibody (left panel), β-synemin (center panel), and the merged image (right panel). Bar = 50 μm

To determine if β-synemin and α-dystrobrevin interact *in vivo*, co-immunoprecipitation experiments were performed from rat muscle homogenates prepared 21 days after the cardiotoxin injection. At this developmental stage, both β-synemin (Fig. 1A, lane 1) and α-dystrobrevin (Fig. 1B, lane 1) are expressed. Using an anti-α-dystrobrevin antibody, β-synemin co-immunoprecipitated and was detectable by western blot analysis (Fig. 1A, lane 2) and was not present in the supernatant after immunoprecipitation (Fig. 1A, lane 3). Similarly, using an anti-β-synemin antibody, both α-dystrobrevin-1 and -2 co-precipitated reconfirming an interaction between β-synemin and α-dystrobrevin (Fig. 1B, lane 2). However, no α-dystrobrevin-1 remained in the supernatant after immunoprecipitation with the anti-β-synemin antibody, while some of the α-dystrobrevin-2 protein did (Fig. 1B, lane 3). These results suggest that the interaction between β-synemin and α-dystrobrevin-1 is stronger than that between β-synemin and α-dystrobrevin-2.

To examine the co-localization of α-dystrobrevin and β-synemin *in vivo*, double immunostaining was performed using the rat tibialis anterior muscle sectioned 21 days after cardiotoxin injection. When the section was stained with an anti-α-dystrobrevin antibody, the muscle cell membrane was homogeneously immunostained (Fig. 1C, left panel) whereas β-synemin was irregularly immunopositive along the sarcolemma in addition to the cytoplasm (Fig. 1C, center panel). Merging the two images confirms that they are co-localized, at least where β-synemin is expressed at the sarcolemma (Fig. 1C, right panel). Although α-dystrobrevin is expressed at the sarcolemma like dystrophin, staining also suggests some expression in the cytoplasm (Fig. 1C, right panel).

**Figure 2:**
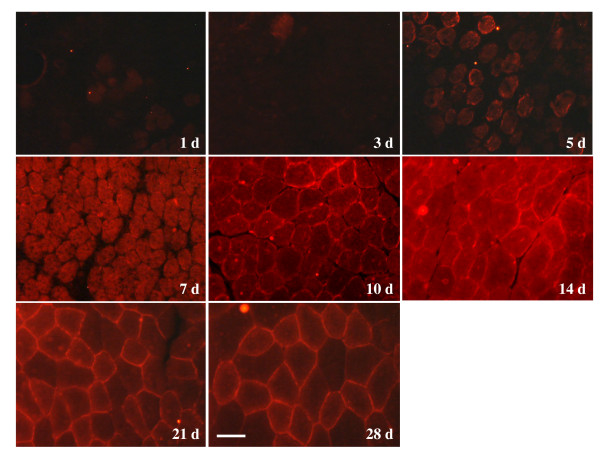
Immunohistochemistry of β-synemin in rat skeletal muscle after injection of cardiotoxin. For the first three days following injection, β-synemin was not detected in transverse muscle sections. β-synemin expression increased from day 5 up to day 14 although sarcolemmal expression appeared to be more prominent on days 21 and 28. Panels are labeled with regards to the number of days (d) following cardiotoxin injection. Bar = 50 μm

Expression patterns for dystrophin, α1-syntrophin, α-dystrobrevin-1, neuronal nitric oxide synthase, α-sarcoglycan, and β-dystroglycan have been reported for regenerating rat muscle [10-13]. By using this same model, it is possible to directly correlate β-synemin's expression with other muscle proteins in regenerating and developing muscle to better understand β-synemin's role in muscle. Cardiotoxin injection is a well-established method to assay muscle regeneration in adult tissue. Using this assay, it is possible to identify proteins that are activated in response to injury. We injected cardiotoxin into the right tibialis anterior muscle in 24 rats. As a control, contralateral muscles in the same animals were injected with sterile 0.9% saline. Muscle was collected at times between days 1 through 28 following injection. Regenerating tissue was excised from the injected rats and examined immunohistochemically to assay β-synemin's and α-dystrobrevin's expression following injury. For the first 3 days following cardiotoxin injection, β-synemin expression was not observed either at the sarcolemma or within the cytoplasm (Fig. 2). On the 5th day, smaller regenerating muscles showed partial sarcolemmal and cytoplasmic staining for β-synemin. Cytoplasmic staining was evident by the 7th day, whereas sarcolemmal staining became more prevalent on the 10th day. The highest level of β-synemin expression was recorded on the 14th day and mosaic sarcolemmal staining was apparent on the 21st and 28th days. With regard to the saline injection control, the muscle was not damaged and showed no changes in β-synemin expression over time (data not shown). In addition, sarcolemmal staining appeared to be fiber specific as some fibers stained both inside and at the sarcolemma much stronger for β-synemin than others.

**Figure 3:**
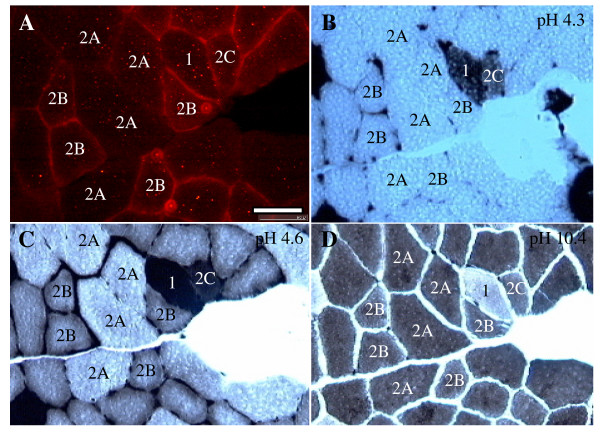
Immunohistochemistry of β-synemin and ATPase staining on muscle sections after injection with cardiotoxin. (A) 10 μm transverse muscle sections were immunostained with an antibody against β-synemin. (B-D) ATPase staining on serial sections was performed at pH 4.3 (B), pH 4.6 (C), and pH 10.4 (D). Type 1 fibers (labeled with a "1") show strong staining at pH 4.3 and pH 4.6 and no staining at pH 10.4, whereas type 2A and 2B fibers (labeled with a "2A" and "2B") show the reverse. Type 2A fibers show weaker staining than type 2B at pH 4.3 and pH 4.6. In addition, type 2C fibers reacted at pH 4.3, pH 4.6, and pH 10.4 and are under the process of regeneration. Bar = 50 μm

It becomes possible to distinguish between different muscle fiber types in regenerating rat muscle after fourteen days following cardiotoxin injection [13]. To determine if β-synemin's mosaic staining pattern was dependent on muscle fiber type, ATPase staining was performed for muscle sectioned 21 and 28 days post-cardiotoxin injection. At 21 days post-injection, β-synemin was expressed at the sarcolemmal membrane in type 1 and type 2C muscle fibers (data not shown). At 28 days post-injection, β-synemin was expressed at the sarcolemmal membrane in type 1, type 2B, and type 2C muscle fibers whereas type 2A fibers did not show sarcolemmal staining (Fig. 3).

**Figure 4:**
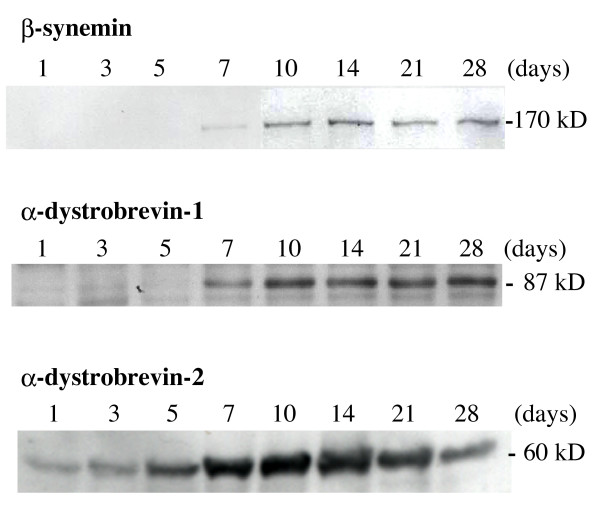
Immunoblot analysis for β-synemin, α-dystrobrevin-1 and -2 following the injection of cardiotoxin. β-Synemin and α-dystrobrevin-1 were detected after day 7, whereas α-dystrobrevin-2 was observed as early as day 1. To normalize for expression levels, immunoblots for β-synemin, α-dystrobrevin-1 and -2 are shown from representative, but different experiments. Approximate molecular weights are indicated on the right.

To better quantitate β-synemin expression during muscle regeneration, immunoblot analysis was performed for muscles collected at different time points following cardiotoxin injection. In agreement with expression data from immunohistochemistry experiments (Fig. 2), β-synemin was detected by immunoblot assay as a weak band on day 7 following cardiotoxin injection. By immunoblot analysis, β-synemin expression increased sequentially for the first 14 days, but thereafter remained almost constant through day 28 (Fig. 4, top panel). Similarly, α-dystrobrevin-1 was first detected on day 7 and its expression remained constant through day 28 (Fig. 4, middle panel). In contrast, α-dystrobrevin-2 expression was apparent as early as 1 day post-injection, and reached maximal expression by day 10, almost 4 days earlier than β-synemin or α-dystrobrevin-1. In addition, α-dystrobrevin-2's expression decreased after day 10 (Fig. 4, bottom panel) unlike α-dystrobrevin-1 and β-synemin.

**Figure 5:**
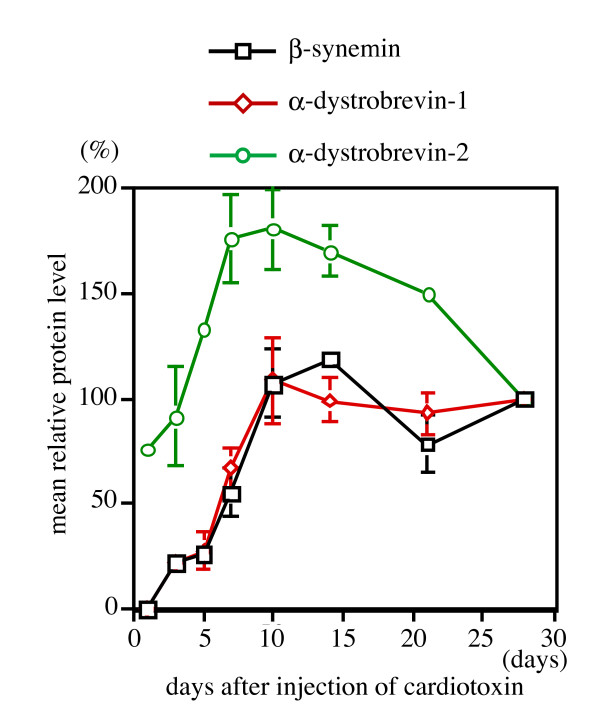
Quantitation of β-synemin, α-dystrobrevin-1 and -2 expression following cardiotoxin injection. The mean relative protein levels were determined for β-synemin, α-dystrobrevin-1 and -2 (n = 9) at each time point by quantifying expression on western blots. The mean relative protein level of β-synemin and α-dystrobrevin-1 on days 1 and 28 were set at 0 and 100%, respectively. α-Dystrobrevin-2 expression at day 1 was not set at 0% because this protein was clearly expressed at that time. For other days, protein levels were calculated by dividing the density of the assayed protein band by the density of the band on day 28 and adjusting the data to a percentage.

To better quantitate these data, immunoblot analysis for β-synemin, α-dystrobrevin-1 and -2 was densitometrically analyzed for each time point and the relative mean protein level for each day was plotted (Fig. 5). β-synemin and α-dystrobrevin-1 reached 50% of their 28 day's expression level by days 6.6 and 6.2, respectively. By day 1, α-dystrobrevin-2 was already at 75% of its expression level relative to that expressed at 28 days. The relative protein expression level for α-dystrobrevin-2 reached its highest point on day 10 at 175% whereupon it started to decline. These differences suggest that α-dystrobrevin-1 and-2 serve different functions in regenerating muscle. Since β-synemin's expression following injury more closely correlates with α-dystrobrevin-1, a protein known to interact with β-synemin *in vitro*, it suggests that these two proteins are more likely to coordinately function *in vivo *than β-synemin and α-dystrobrevin-2.

**Figure 6:**
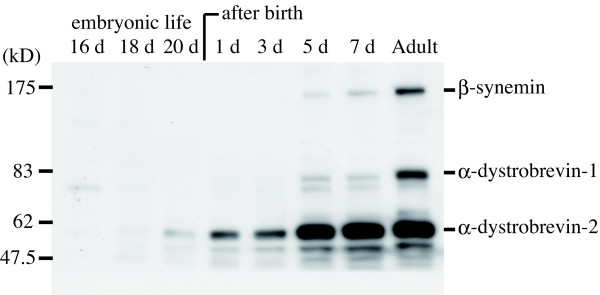
β-synemin, α-dystrobrevin-1 and -2 are predominantly expressed after birth. Lower-limb muscles from rats at embryonic day 16 through 7 days post-birth were harvested and analyzed using an immunoblot assay. β-synemin and α-dystrobrevin-1 were first detected 5 days after birth, while α-dystrobrevin-2 was expressed in day 20 embryos.

To investigate if protein expression patterns for β-synemin, α-dystrobrevin-1 and α-dystrobrevin-2 are similar between muscle regeneration and development, lower-limb muscles from rats at embryonic day 16 through 7 days post-birth were examined (Fig. 6). β-synemin and α-dystrobrevin-1 were first expressed at 5 days post-birth, although even then, expression was significantly less than that of adult muscle (Fig. 6). In contrast, α-dystrobrevin-2 expression was seen before birth in 20-day-old embryos. Expression of α-dystrobrevin-2 quickly increased such that α-dystrobrevin-2 reached adult levels by 7 days post-birth whereas β-synemin and α-dystrobrevin-1 were below adult levels. Similar to that shown for regenerating muscle, these data from developing muscle show that β-synemin expression better correlates with α-dystrobrevin-1 than α-dystrobrevin-2 suggesting that β-synemin was more likely to function concordantly with α-dystrobrevin-1 *in vivo*.

## Discussion

Mammalian β-synemin was originally identified as an α-dystrobrevin-binding protein through a yeast two-hybrid screen using an amino acid sequence derived from exons 1 through 16 of α-dystrobrevin [2,14], a region common to both α-dystrobrevin-1 and -2. Therefore, it is unknown from this analysis, whether each α-dystrobrevin isoform has the same affinity for β-synemin or whether one of the isoforms functions coordinately with β-synemin *in vivo*. Muscle degeneration and regeneration can be induced by injecting bupivacaine [15], the venom of Australian tiger snake [16], and cardiotoxin [10-13,17,18] from the Taiwan cobra (Naja naja atra) directly into rat muscle. Using this assay, we analyzed β-synemin and α-dystrobrevin to correlate their expression patterns during skeletal muscle regeneration.

Previously, we had reported that β-synemin expression was normal in dystrophin-deficient (mdx) muscle [4]. Whereas α-dystrobrevin-2 was greatly reduced in mdx muscle, α-dystrobrevin-1 was unaffected [4] suggesting that β-synemin may preferentially interact with that α-dystrobrevin isoform *in vivo*. In addition, α-dystrobrevin-1 predominantly localized to the crest of the neuromuscular junctions (NMJs), whereas α-dystrobrevin-2 was expressed at the deep portions of the NMJs [19], suggesting the two α-dystrobrevin isoforms may have very different functions. By determining which isoform of α-dystrobrevin is associated with β-synemin *in vivo*, we can begin to better understand what function β-synemin serves in developing and regenerating muscle.

This study shows that β-synemin and α-dystrobrevin-1 preferentially interact *in vivo*. For example, when α-dystrobrevin was co-immunoprecipitated using an anti-β-synemin antibody, all of α-dystrobrevin-1 immunoprecipitated whereas only approximately half of the α-dystrobrevin-2 isoform did (Fig. 1B). This suggests that the interaction between β-synemin and α-dystrobrevin-1 is stronger than that of β-synemin and α-dystrobrevin-2 although it is possible that different expression levels for the α-dystrobrevin isoforms also could have played some role. In addition, we also show that β-synemin expression strongly correlates with α-dystrobrevin-1 (rather than α-dystrobrevin-2) in rat muscle development and regeneration (Fig. 2, 4, 5, 6). These data strongly suggest that β-synemin associates predominantly with the α-dystrobrevin-1 isoform.

ATPase assays are used to distinguish between muscle fiber types. Type 1 fibers are slow-twitch fibers, whereas type 2A and 2B fibers correspond to fast-red and fast-white fibers, respectively [20]. Previous results have shown that nNOS is preferentially expressed in type 2B fibers at 14 days post-injection in regenerating rat muscle [13]. nNOS is thought to associate with α-dystrobrevin [4], which in turn associates with β-synemin. While this provides a potential explanation for fiber type specific protein expression, the physiological significance of β-synemin's expression in type 1, 2B, and 2C fibers rather than 2A fibers is not fully understood; however, β-synemin's expression might be related to the regulation of fatigue in type 1, 2B, and type 2C fibers.

Confocal microscopic analysis positioned β-synemin to the costamere and muscle Z-lines [4]. In addition, β-synemin was enriched at the neuromuscular and myotendinous junctions [4]. Based on its localization and its expression pattern, β-synemin is thought to function as a structural protein involved in maintaining muscle integrity through its interactions with either α-dystrobrevin or other structural proteins. Our present findings that β-synemin (6.6 day) and α-dystrobrevin-1 (6.2 day) are expressed at similar times in regenerating muscle are similar to that previously reported for α 1-syntrophin (6.0 day) [10], suggesting that these three muscle proteins may be involved in a common pathway. Since α 1-syntrophin and α-dystrobrevin-1 are both involved in known signaling pathways [9,21,22], it is suggestive that β-synemin is also associated with signaling [23].

With regard to the expression patterns of β-synemin and α-dystrobrevin during development, it should be noted that there are instances in which the results reported here are slightly different than that reported in the literature. For example, Xue et al. previously demonstrated that β-synemin was expressed at embryonic day 13 in forelimb and hind limb of mice [24] whereas it was detected only after 5 days post-birth in our study. In addition, Nawrotzki et al. reported that C2 myoblasts and early myotubes express α-dystrobrevin-1 whereas α-dystrobrevin-2 is only expressed in late myotubes [25]. In contrast, we find that α-dystrobrevin-2 is expressed before birth in 20-day-old embryos whereas α-dystrobrevin-1 is expressed only after 5 days post-birth. These differences are hard to reconcile; however, it is possible that (1) species differences played some role or (2) that the different anti-α-dystrobrevin antibodies used in these studies had slightly different specificities.

After cardiotoxin injection, α- dystrobrevin-2 and β-dystroglycan [11] were expressed in muscle as early as 1 day post-injection, while α-sarcoglycan [11], dystrophin [10-13], α 1-syntrophin [10], β-synemin, and α-dystrobrevin-1 [12] were not detected in the rat model until later. Relative to its expression at day 28, β-dystroglycan was expressed at 50% at day 1 and 100% by day 10 [11]. α-Dystrobrevin-2 was expressed at 75% by day 1 and increased to 130% at day 5, indicating that α-dystrobrevin-2 was expressed earlier in regenerating muscle than β-dystroglycan. Since laminin persists in the basal lamina after cardiotoxin injection [26], it is possible that β-dystroglycan, which indirectly binds to laminin-2 through α-dystroglycan, is stabilized by this interaction [11]. It is unknown how or why α-dystrobrevin-2 is expressed in the early stages of muscle regeneration when other sarcolemmal proteins like dystrophin and its associated proteins are not. What is unclear is how α-dystrobrevin-2 evades the effects of cardiotoxin injection, although it is possible that this isoform is somehow stable in the degenerating fibers or that it is expressed in cells other than muscle.

## Conclusion

Our results show that β-synemin and α-dystrobrevin-1 co-localize and preferentially interact *in vivo*. In addition, β-synemin and α-dystrobrevin-1 are highly up-regulated at the same stages during muscle development and repair. This result is in contrast to that of α-dystrobrevin-2 which is expressed much earlier in these myogenic processes. These data strongly suggest that β-synemin is more likely to function with α-dystrobrevin-1 *in vivo *providing insight into the possible roles of these proteins in muscle.

## Abbreviations

ABC, avidin-biotinylated enzyme complex; DAPC, dystrophin-associated protein complex; HRP, horseradish peroxidase; NMJ, neuromuscular junction; nNOS, neuronal nitric oxide synthase; SDS-PAGE, sodium dodecylsulfate-polyacrylamide gel electrophoresis;

## Competing interests

The author(s) declare that they have no competing interests.

## Authors' contributions

All authors read and approved the final manuscript.

## Pre-publication history

The pre-publication history for this paper can be accessed here:
